# Basedow’s disease with associated features of Hashimoto’s thyroiditis based on histopathological findings

**DOI:** 10.1186/s12902-020-00602-8

**Published:** 2020-08-05

**Authors:** Megumi Horiya, Takatoshi Anno, Fumiko Kawasaki, Yuichiro Iwamoto, Shintaro Irie, Yasumasa Monobe, Koichi Tomoda, Kohei Kaku, Shuhei Nakanishi, Hideaki Kaneto

**Affiliations:** 1 Department of General Internal Medicine 1, Kawasaki Medical School, 2-6-1 Nakasange, Kita-ku, Okayama, 700-8505 Japan; 2 Department of Pathology, Kawasaki Medical School, Okayama, 700-8505 Japan; 3 Department of Diabetes, Metabolism and Endocrinology, Kawasaki Medical School, Kurashiki, 701-0192 Japan

**Keywords:** Basedow’s disease, Hashimoto’s thyroiditis, Histopathological features, Autoimmune antibody

## Abstract

**Background:**

Basedow’s disease and Hashimoto’s thyroiditis are autoimmune thyroid disorders and usually diagnosed with elevation of serum autoimmune antibodies. Thyrotropin receptor antibodies (TRAb) and/or thyroid-stimulating antibody (TSAb) are usually used for diagnosis of Basedow’s disease, and thyroid peroxidase antibodies (TPOAb) and/or thyroglobulin antibodies (TgAb) are for diagnosis of Hashimoto’s thyroiditis. However, it is difficult to diagnose a subject as Basedow’s disease with associated features of Hashimoto’s thyroiditis only with elevation of such autoimmune antibodies.

**Case presentation:**

A 44-year-old woman with 5-year history of Basedow’s disease underwent a total thyroidectomy. She did not have a goiter. TRAb, TSAb, TPOAg and TgAb were all positive before a total thyroidectomy. In histopathological macroscopic examination, diffuse hyperplasia of the thyroid gland was observed. Furthermore, in histopathological microscopic examination, both characteristics of Basedow’s disease and Hashimoto’s thyroiditis were observed. After a total thyroidectomy, titers of all thyroid-associated autoimmune antibodies were markedly reduced.

**Conclusion:**

Herein, we report a subject with Basedow’s disease without a goiter whose TPOAb and TgAb were relatively high at the onset of Basedow’s disease. In addition, interestingly, the histopathological findings of this subject showed direct signs of Basedow’s disease and Hashimoto’s thyroiditis in the same thyroid gland. Considering from such findings, she seemed to have Basedow’s disease with associated features of Hashimoto’s thyroiditis. In conclusion, we should bear in mind the possibility of Basedow’s disease with associated features of Hashimoto’s thyroiditis in subjects with Basedow’s disease, particularly when TPOAb and TgAb as well as TRAb and TSAb are positive.

## Background

Basedow’s disease and Hashimoto’s thyroiditis are autoimmune diseases of the thyroid gland. Basedow’s disease is the most common cause of hyperthyroidism. On the other hand, Hashimoto’s thyroiditis, which is also known as chronic lymphocytic thyroiditis, shows various levels of thyroid hormones. For example, it shows hypothyroidism when the thyroid gland is gradually destroyed by antibody-mediated autoimmune process.

The thyroid gland often becomes hypervascular in subjects with Basedow’s disease, and thus it is difficult to perform thyroid gland biopsy. However, once thyroid gland biopsy or thyroidectomy is performed, histopathological features of Basedow’s disease are proliferation of follicular components, tall columnar thyroid epithelium cells, hyperplastic infoldings into the colloid and clear vacuole change in the colloid. In contrast, histopathological features of Hashimoto’s thyroiditis usually consist of lymphoplasmacytic infiltration and lymphoid follicle formation with well-developed germinal centers. However, Hashimoto’s thyroiditis is not a histopathological homogeneous lesion. Thus, Basedow’s disease and Hashimoto’s thyroiditis used to be considered as distinct entities.

Furthermore, autoimmune antibodies are important for diagnosis of Basedow’s disease and Hashimoto’s thyroiditis. Both diseases are autoimmune disorders, and usually diagnosed with elevation of various serum autoimmune antibodies. Thyrotropin receptor antibodies (TRAb) and/or thyroid stimulating antibody (TSAb) are usually used for diagnosis of Basedow’s disease [1, 2], and thyroid peroxidase antibodies (TPOAb) and/or thyroglobulin antibodies (TgAb) are for diagnosis of Hashimoto’s thyroiditis [3, 4]. However, seronegative thyroiditis (without any circulating autoantibodies) is also sometimes observed [5]. In addition, TPOAb and TgAb are elevated in some subjects with Basedow’s disease as well as Hashimoto’s thyroiditis [6] which makes it difficult to diagnose these two diseases. It has been proposed recently that there might be some continuity between Basedow’s disease and Hashimoto’s thyroiditis [7, 8].

Herein, we report a case showing Basedow’s disease with associated features of Hashimoto’s thyroiditis in histopathological findings. Titers of TRAb, TSAb, TPOAb and TgAb were very high before a total thyroidectomy. Interestingly, in histopathological examination, her thyroid specimen showed both characteristics of Basedow’s disease and Hashimoto’s thyroiditis. In addition, after a thyroidectomy all autoimmune antibodies were markedly decreased. These data suggest that she suffered from Basedow’s disease with associated features of Hashimoto’s thyroiditis.

## Case presentation

**Fig. 1:**
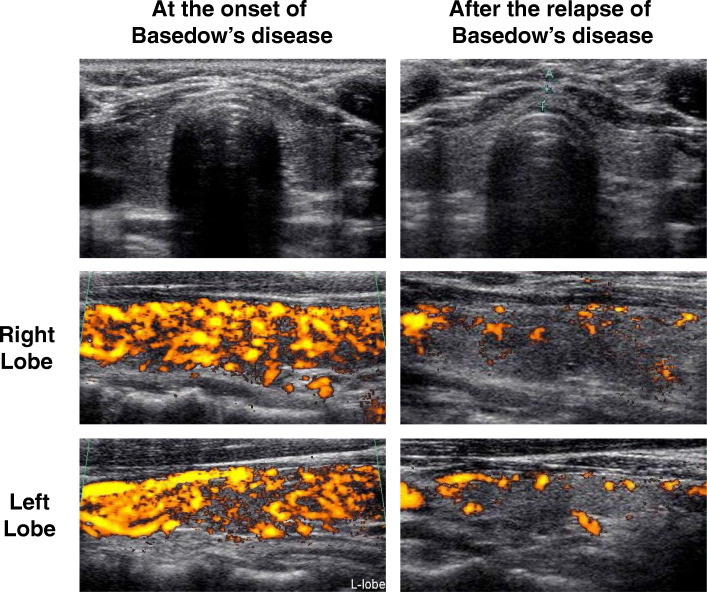
Ultrasound examination at the onset of Basedow’s disease revealed that the thyroid gland was hypervascular although it was not enlarged. Ultrasound examination after the relapse of Basedow’s disease revealed that the thyroid gland was not very hypervascular compared to that at the onset of the disease

A 44-year-old woman with 5-year history of Basedow’s disease had a total thyroidectomy. She had no past and family history and had no drug allergy. She was diagnosed as Basedow’s disease at 39 years old and after then she started taking 30 mg of thiamazole (MMI). In physical examination, she had no remarkable symptom such as palpitation, general fatigue and insomnia and did not have a goiter. Her height and body weight were 158.0 cm and 63.6 kg. Her vital signs were: heart rate 112 beats/min, blood pressure 132/86 mmHg. Laboratory data were as follows: white blood cell count, 4580 /μL (neutrophil 57.7%); red blood cell count, 476 × 10^4^ /μL; hemoglobin, 12.7 g/dL; platelet, 26.5 × 10^4^ /μL; Na, 142 mmol/L; K, 5.2 mmol/L. Renal and liver function was within normal range (creatinine (CRE), 0.38 mg/dL; blood urea nitrogen (BUN), asparate aminotransferase (AST), 25 U/L; alanine transaminase (ALT), 26 U/L; alkaline phosphatase (ALP), 231 U/L; γ-glutamyltranspeptidase (γ-GTP), 17 U/L; lactate dehydrogenase (LDH), 174 U/L). Thyroid-associated data were as follows: thyroid-stimulating hormone (TSH), < 0.010 μIU/mL; free triiodothyronine (FT3), 19.05 pg/mL; free thyroxine (FT4) 4.88 ng/dL; TRAb, 10.6 IU/L (electro chemiluminescence immunoassay (ECLIA), SRL Inc., Tokyo); TPOAb, 216.9 IU/mL (ECLIA, SRL Inc., Tokyo); TgAb antibody, 428.9 IU/mL (ECLIA, SRL Inc., Tokyo). Ultrasound examination revealed that the thyroid gland was hypervascular although it was not enlarged (Fig. 1). Based on such findings, we finally diagnosed her as Basedow’s disease. Two weeks after starting MMI therapy, she had liver dysfunction (AST, 420 U/L; ALT, 368 U/L; ALP, 565 U/L; γ-GTP, 178 U/L; LDH, 337 U/L), and we changed the treatment of MMI to 300 mg of propylthiouracil (PTU) although her thyroid hormone levels were decreased (TSH, < 0.010 μIU/mL; FT3, 7.2 4 pg/mL; F T4 1.97 ng/dL). After then, her liver function was improved, and we tapered PTU. About 2 years later, her thyroid hormone levels were within normal range with 50 mg of PTU every other day, and her TRAb became negative. After then, we stopped PTU therapy.

About 1.5 years later, she had low grade fever and palpitation and visited our office again. Her body weight was 70.9 kg. Her vital signs were: heart rate 116 beats/min, blood pressure 140/98 mmHg. Laboratory data were as follows: white blood cell count, 3710 /μL (neutrophil 58.3%); red blood cell count, 535 × 10^4^ /μL; hemoglobin, 12.2 g/dL; platelet, 22.4 × 10^4^ /μL; Na, 142 mmol/L; K, 4.2 mmol/L. Renal and liver function was within normal range (CRE, 0.52 mg/dL; BUN 9 mg/dL; AST, 25 U/L; ALT, 17 U/L; ALP, 218 U/L; γ-GTP, 19 U/L; LDH, 206 U/L). Thyroid-associated data were as follows: TSH, < 0.010 μIU/mL; FT3, 5.22 pg/mL; FT4, 1.85 ng/dL; TRAb, 4.6 IU/L; TPOAb, 147.4 IU/mL; TgAb, 591.2 IU/mL. Ultrasound examination revealed that the thyroid gland was not hypervascular compared to that at the onset of Basedow’s disease (Fig. 1). Based on such findings, we finally diagnosed her as the relapse of Basedow’s disease. We started again 300 mg of PTU and tapered PTU dose. About 4 months later, her thyroid hormone levels became within normal range by taking 50 mg of PTU every other day. During the PTU therapy, her WBC levels were slightly lower and CRP levels were slightly higher, although she had no symptom in the whole body including in skin. We checked various autoimmune antibodies. Anti-double stranded DNA IgG antibody, anti-SS-A/Ro antibody and anti-SS-B/La antibody were all negative. However, anti-nuclear antibody (ANA), myeloperoxidase-anti-neutrophil cytoplasmic antibody (MPO-ANCA) and proteinase3-anti-neutrophil cytoplasmic antibody (PR3-ANCA) were all positive (ANA, 37.8 (+); MPO-ANCA, 35.0 U/mL; PR3-ANCA, 36.9 U/mL).

**Fig. 2:**
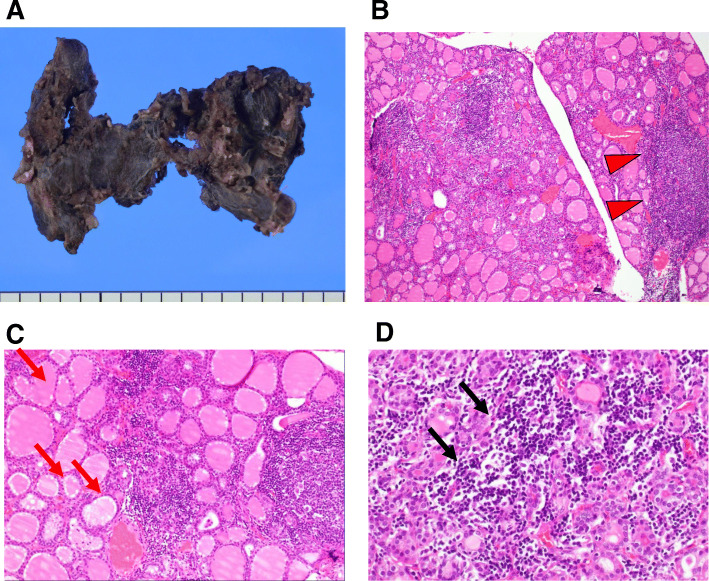
A. Histopathological macroscopic findings. The thyroid gland was flat and atrophic although its area was wide. B.C.D. Histopathological microscopic findings. Diffuse hyperplasia of the thyroid gland and hyperplastic thyroid follicles with papillary infoldings was observed on histopathological image. Tall follicular cells with papillae lacked fibrovascular cores and peripheral scalloping was present (red arrow). On the other hand, a part of the thyroid area showed a dense lymphoplasmacytic infiltrate, accompanied by follicles containing germinal centers (red triangle). In addition, a destroyed pattern of lymphoid follicle was observed (black arrow)

We performed a total thyroidectomy. Table 1 shows laboratory data before a total thyroidectomy in this subject taking 50 mg of PTU every other day. In general, the thyroid gland in subjects with Basedow’s disease shows diffuse goiter due to chronic stimulation. However, her thyroid was flat and atrophic although diffuse hyperplasia of the thyroid gland was observed in histopathological macroscopic findings (Fig. 2A). Furthermore, hyperplasia of the thyroid gland was shown in part and hyperplastic thyroid follicles with papillary infoldings was observed in histopathological microscopic examination. Tall follicular cells with papillae usually lacked fibrovascular cores and peripheral scalloping was present (Fig. 2B, C). On the other hand, a part of the thyroid area showed a dense lymphoplasmacytic infiltrate, accompanied by follicles containing germinal centers. In addition, a destroyed pattern of lymphoid follicle was observed (Fig. 2B, D).

**Table 1:** Laboratory data before total thyroidectomy in this subject

Variable	Result	Reference range	Variable	Result	Reference range
Peripheral blood			Dyslipidemia marker		
White blood cells (/μL)	3340	3300–8600	Total cholesterol (mg/dL)	229	142–248
Red blood cells (× 10^4^/μL)	496	435–555	LDL cholesterol (mg/dL)	136	65–139
Hemoglobin (g/dL)	11.0	13.7–16.8	HDL cholesterol (mg/dL)	76	40–90
Platelets (× 10^4^/μL)	29.0	15.8–34.8	Triglyceride (mg/dL)	66	40–149
Blood biochemistry			Thyroid marker		
Total protein (g/dL)	7.2	6.6–8.1	TSH (μIU/mL)	0.010	0.400–6.000
Albumin (g/dL)	3.8	4.1–5.1	FT3 (pg/mL)	3.92	2.50–4.20
Globulin (g/dL)	3.4	2.2–3.4	FT4 (ng/dL)	0.97	0.80–1.60
Total bilirubin (mg/dL)	0.5	0.4–1.5	Thyroglobulin (ng/mL)	0.59	0.00–33.70
AST (U/L)	18	13–30	TRAb (IU/L)	5.4	< 1.0
ALT (U/L)	16	10–42	TSAb (%)	132	0–120
LDH (U/L)	195	124–222	TPOAb (IU/mL)	98.3	< 16.0
ALP (U/L)	143	106–322	TgAb (IU/mL)	151.0	< 28.0
γ-GTP (U/L)	10	13–64	ANA	37.8 (+)	< 20.0
BUN (mg/dL)	9	8–20	Anti-ds-DNA Ab (IU/mL)	< 10	0–12
Creatinine (mg/dL)	0.60	0.65–1.07	anti-SS-A/Ro Ab (U/mL)	1	< 10.0
Cholinesterase (U/L)	292	240–486	anti-SS-B/La Ab (U/mL)	6.4	< 10.0
Uric acid (mg/dL)	3.5	2.6–5.5	MPO-ANCA (U/mL)	35.0	< 3.5
Creatine Kinase (U/L)	43	41–153	PR3-ANCA (U/mL)	36.9	< 3.5
Amylase (U/L)	95	42–118	Intact PTH (pg/mL)	30	10–65
CRP (mg/dL)	0.32	< 0.14	Urinary test		
Sodium (mmol/L)	133	138–145	Urinary pH	5.0	5.0–7.5
Potassium (mmol/L)	5.5	3.6–4.8	Urinary protein	–	–
Chloride (mmol/L)	100	101–108	Urinary sugar	–	–
IP (mg/dL)	2.7	2.7–4.6	Urinary ketone body	–	–
Calcium (mg/dL)	8.9	8.8–10.0	Urinary bilirubin	–	–
Plasma glucose (mg/dL)	115		Urinary blood	–	–
Hemoglobin A1c (%)	5.7	4.9–6.0			

Abbreviation: AST, aspartate aminotransferase; ALT, alanine aminotransferase; LDH, lactate dehydrogenase; ALP, alkaline phosphatase; γ-GTP, γ-glutamyltranspeptidase; BUN, blood urea nitrogen; CRP, C-reactive protein; IP, Inorganic Phosphorus; LDL, Low-density lipoprotein; HDL, High-density lipoprotein

TSH, thyroid stimulating hormone; FT3, free triiodothyronine; free thyroxine; FT4; TRAb, thyrotropin receptor; TSAb, thyroid stimulating antibody; TPOAb, anti-thyroid peroxidase antibodies; TgAb, anti-thyroglobulin antibody; ANA, anti-nuclear antibody; Anti-ds-DNA Ab, anti-double stranded DNA IgG antibody; MPO-ANCA, myeloperoxidase-anti-neutrophil cytoplasmic antibody; PR3-ANCA, proteinase3-anti-neutrophil cytoplasmic antibody; PTH, parathyroid hormone

One year later, thyroid-associated autoimmune antibodies were all decreased (TRAb, 2.2 IU/L; TSAb, 130% (enzyme immunoassay, SRL Inc., Tokyo); TPOAb, 98. 3 IU/mL; TgAb, 151.0 IU/mL), and 2 years later thyroid-associated autoimmune antibodies were further improved (TRAb, 1.1 IU/L; TSAb, 90%; TPOAb, 24.0 IU/mL; TgAb, 12.8 IU/mL). White blood cell count became within normal range (about 5000–7000 /μL (neutrophil 58–68%) and CRP level became almost normal (0.15–0.20 mg/dL).

## Discussion and conclusions

In this report, we showed a case of Basedow’s disease with associated features of Hashimoto’s thyroiditis, which was diagnosed with histopathological features after a total thyroidectomy. It is sometimes difficult to diagnose these two diseases, because TPOAb and TgAb are elevated in Basedow’s disease as well as Hashimoto’s thyroiditis. It is thought that Basedow’s disease and Hashimoto’s thyroiditis are most common thyroid-specific autoimmune diseases and are closely related to each other from the point of pathophysiological process. The onset of Basedow’s disease or Hashimoto’s thyroiditis are influenced by balance of Th1/Th2 cytokines and it seems that increase of the Th1/Th2 cell ratio induces Hashimoto’s disease rather than Basedow’s disease [9, 10]. There are a few reports showing the transformation of Hashimoto’s thyroiditis to Basedow’s disease [11–15], but in many of these reports such arguments are based on clinical and laboratory features.

At first, we diagnosed her as Basedow’s disease and started therapy with MMI and PTU. In addition, since her laboratory data showed slightly high CRP levels, we checked ANA level, MPO-ANCA and PR3-ANCA after obtaining euthyroid with PTU therapy. We performed a total thyroidectomy. After then, TRAb, TSAb, TPOAb and TgAb were markedly decreased. In addition, Basedow’s disease is often treated with anti-thyroid drugs. However, risk for relapse is relatively high and patients may experience some side effects [16]. In patients with normalized TRAb, relapse rate is 20 to 30% over 3 to 5 years in follow-up period [17]. TPOAb or TgAb changes during anti-thyroid drug treatment was also reported [18]. She experienced relapse of Basedow’s disease and had histopathological features of both Basedow’s disease and Hashimoto’s thyroiditis. Based on such findings, we finally diagnosed her as having Basedow’s disease and Hashimoto’s thyroiditis. As described above, ANCA antibodies were positive in this subject. Since it is well known that ANCA-associated vasculitis is an autoimmune disease which can affect multiple organs, we cannot exclude the possibility that ANCA antibodies are, at least in part, associated with the coexistence of Basedow’s disease and Hashimoto’s thyroiditis, but further study would be necessary to elucidate this point.

It is controversial how Basedow’s disease is complicated with Hashimoto’s thyroiditis and in clinical practice it is difficult to diagnose a subject as Basedow’s disease together with Hashimoto’s thyroiditis. Hashitoxicosis, which is known to be the initial hyperthyroid phase in chronic autoimmune thyroiditis, shows histopathological findings of both Basedow’s disease and Hashimoto’s thyroiditis [19]. Hashitoxicosis is often misdiagnosed with acute exacerbation of Hashimoto’s thyroiditis, because it is sometimes difficult to distinguish these two diseases. Interestingly, her thyroid gland showed both histopathological features of Basedow’s disease and Hashimoto’s thyroiditis, although her main physiological findings were compatible with Basedow’s disease. In macroscopic examination, her thyroid gland was flat and atrophic although its area was large. It was different from typical thyroid gland in subjects with Basedow’s disease showing diffuse goiter. In addition, in microscopic examination, diffuse hyperplasia of the thyroid gland and hyperplastic thyroid follicles with papillary infoldings were observed. Tall follicular cells with papillae lacked fibrovascular cores and peripheral scalloping was present. In general, these findings are observed in Basedow’s disease. On the other hand, some part of thyroid area showed a dense lymphoplasmacytic infiltrate, accompanied by follicles containing germinal centers. In addition, a destroyed pattern of lymphoid follicle was also observed. In general, these findings are observed in Hashimoto’s thyroiditis, although they are typically diffuse but scattered area. These microscopic findings were similar to those with Hashitoxicosis, and based on such histopathological findings, we diagnosed her as Basedow’s disease with associated features of Hashimoto’s thyroiditis.

There are several strengths in this case report. First, this patient had a typical course of Basedow’s disease at first, although she did not have a goiter. At the onset of Basedow’s disease, her TPOAb and TgAb were relatively high. Considering from such findings, she seemed to have Basedow’s disease with associated features of Hashimoto’s thyroiditis, but it was difficult to precisely diagnose this subject only with symptom and thyroid-associated autoimmune antibodies. Second, there is few reports clearly showing Basedow’s disease with associated features of Hashimoto’s thyroiditis. TPOAb and TgAb are sometimes detected in subjects with Basedow’s disease which raises the possibility of coexistence of Basedow’s disease and Hashimoto’s thyroiditis. However, it has not been reported so far about the literal coexistence of Basedow’s disease and Hashimoto’s thyroiditis. In this report, we showed that this subject had Basedow’s disease with associated features of Hashimoto’s thyroiditis based on histopathological findings. Interestingly, such findings showed direct histological signs of Basedow’s disease and Hashimoto’s thyroiditis in the same thyroid gland.

There are also several limitations in this report. First, since we performed a total thyroidectomy at the onset of Basedow’s disease, the precise pathogenesis in this subject remained unknown. Second, since we did not perform radionuclide imaging in this patient, we failed to demonstrate an expected pattern of diffusely increased uptake which is often observed in Basedow’s disease. We think that it would be interesting to know what happens if this subject continues to take anti-thyroid drugs without taking an operation. It would be possible that continuous treatment would lead to amelioration of thyroid function which possibly accompanied by mitigation of hyper-vascularization. Furthermore, it would be intriguing to know whether or not such alteration could influence the histopathological findings which are characteristics of Hashimoto’s thyroiditis.

Taken together, we should bear in mind the possibility of Basedow’s disease with associated features of Hashimoto’s thyroiditis in histopathological findings among subjects with Basedow’s disease, particularly when not only TRAb and TSAb but also TPOAb and TgAb are positive.

## Data Availability

Not applicable.

## Abbreviations

TRAb: thyrotropin receptor antibodies
TSAb: thyroid-stimulating antibody
TPOAb: thyroid peroxidase antibodies
TgAb: thyroglobulin antibodies
MMI: thiamazole
CRE: Creatinine
BUN: blood urea nitrogen
AST: asparate aminotransferase
ALT: alanine transaminase
ALP: alkaline phosphatase
(ALP) γ-GTP: γ-glutamyltranspeptidase
LDH: lactate dehydrogenase
TSH: thyroid-stimulating hormone
FT3: free triiodothyronine
FT4: free thyroxine
PTU: propylthiouracil
ANA: anti-nuclear antibody
MPO-ANCA: myeloperoxidase-anti-neutrophil cytoplasmic antibody
PR3-ANCA: proteinase3-anti-neutrophil cytoplasmic antibody
